# Recombinant FOXN1 fusion protein increases T cell generation in old mice

**DOI:** 10.3389/fimmu.2024.1423488

**Published:** 2024-07-12

**Authors:** Jin Zhao, Rong Hu, Kuan Chen Lai, Zhenzhen Zhang, Laijun Lai

**Affiliations:** ^1^ Department of Allied Health Sciences, University of Connecticut, Storrs, CT, United States; ^2^ Institute for Systems Genomics, University of Connecticut, Storrs, CT, United States; ^3^ University of Connecticut Stem Cell Institute, University of Connecticut, Storrs, CT, United States

**Keywords:** FOXN1, thymus, thymic epithelial cells, T cell generation, old mice

## Abstract

T cell development in the thymus is dependent on the thymic microenvironment, in which thymic epithelial cells (TECs) are the major component. However, TECs undergo both a qualitative and quantitative loss during aging, which is believed to be the major factor responsible for age-dependent thymic atrophy. FOXN1 plays a critical role in TEC development and adult TECs maintenance. We have previously reported that intrathymic injection of a recombinant (r) protein containing murine FOXN1 and a protein transduction domain increases the number of TECs in mice, leading to enhanced thymopoiesis. However, intrathymic injection may not be an ideal choice for clinical applications. In this study, we produced a rFOXN1 fusion protein containing the N-terminal of CCR9, human FOXN1 and a protein transduction domain. When injected intravenously into 14-month-old mice, the rFOXN1 fusion protein enters the thymus and TECs, and enhances thymopoiesis, resulting in increased T cell generation in the thymus and increased number of T cells in peripheral lymphoid organ. Our results suggest that the rFOXN1 fusion protein has the potential to be used in preventing and treating T cell immunodeficiency in older adults.

## 1 Introduction

Aging affects several organ systems, and the immune system is one of the systems most significantly affected (1, 2). The deleterious effects of aging on the immune system are well recognized as they lead to increased susceptibility to infection and cancer. In addition, the efficiency of vaccination is significantly reduced, limiting preventative prophylaxis.

The thymus is a specified immune organ that provides an inductive environment for the generation of T cells that play a critical role in the adaptive immune system. Although the thymus continues to export T cells throughout life, it undergoes a profound atrophy with age, a process termed thymic involution, resulting in decreased numbers and functional capacity of T cells in older adults, which has direct etiological linkages with many diseases (3–5). Furthermore, T cell immune deficiency in the older adult is exacerbated when the immune system is insulted by chemotherapy, radiotherapy, infections (e.g. HIV), and preparative regimens for foreign tissue or organ transplants. Therefore, restoring thymus function in the older adult has important implications (3–5).

T cell development in the thymus is dependent on the thymic microenvironment, in which thymic epithelial cells (TECs) are the major component (6–10). Despite their importance, TECs undergo both a qualitative and quantitative loss over time, which is believed to be the major factor responsible for age-dependent thymic atrophy/involution (3–5). It is generally acknowledged that FOXN1 is a critical regulator for TEC development (11–17). FOXN1 is expressed in both fetal thymus and postnatal TECs and its expression is downregulated with aging (18–23). FOXN1 is required for TEC development in fetal thymus and the maintenance of the postnatal thymus (17–21, 24, 25).

We have previously reported that intrathymic injection (i.t.) of a recombinant (r) protein containing murine FOXN1 and a protein transduction domain embedded in the HIV transactivator of transcription (TAT) protein (amino acids 47–57) increases the number of TECs in mice that have undergone congenic or allogeneic hematopoietic stem cell transplantation (26) and mice with Alzheimer’s disease (27). Consequently, these mice had enhanced thymopoiesis, an improved thymic output and an increased number of naïve T cells in the periphery (26). However, i.t. injection may not be an ideal choice for clinical applications.

It has been reported that chemokine CCL25 is highly expressed in thymic tissue, especially thymic stroma (28). CCR9 is the receptor for CCL25 (29). Unlike other CC chemokine receptors, CCR9 shows a strict specificity for its ligand CCL25 (28–30). It has been shown intramural injection of a fusion protein containing the N-terminal of CCR9 and IL-7 increased the content of IL-7 in the thymus as compared to injection of IL-7 alone (28).

**Scheme 1:**
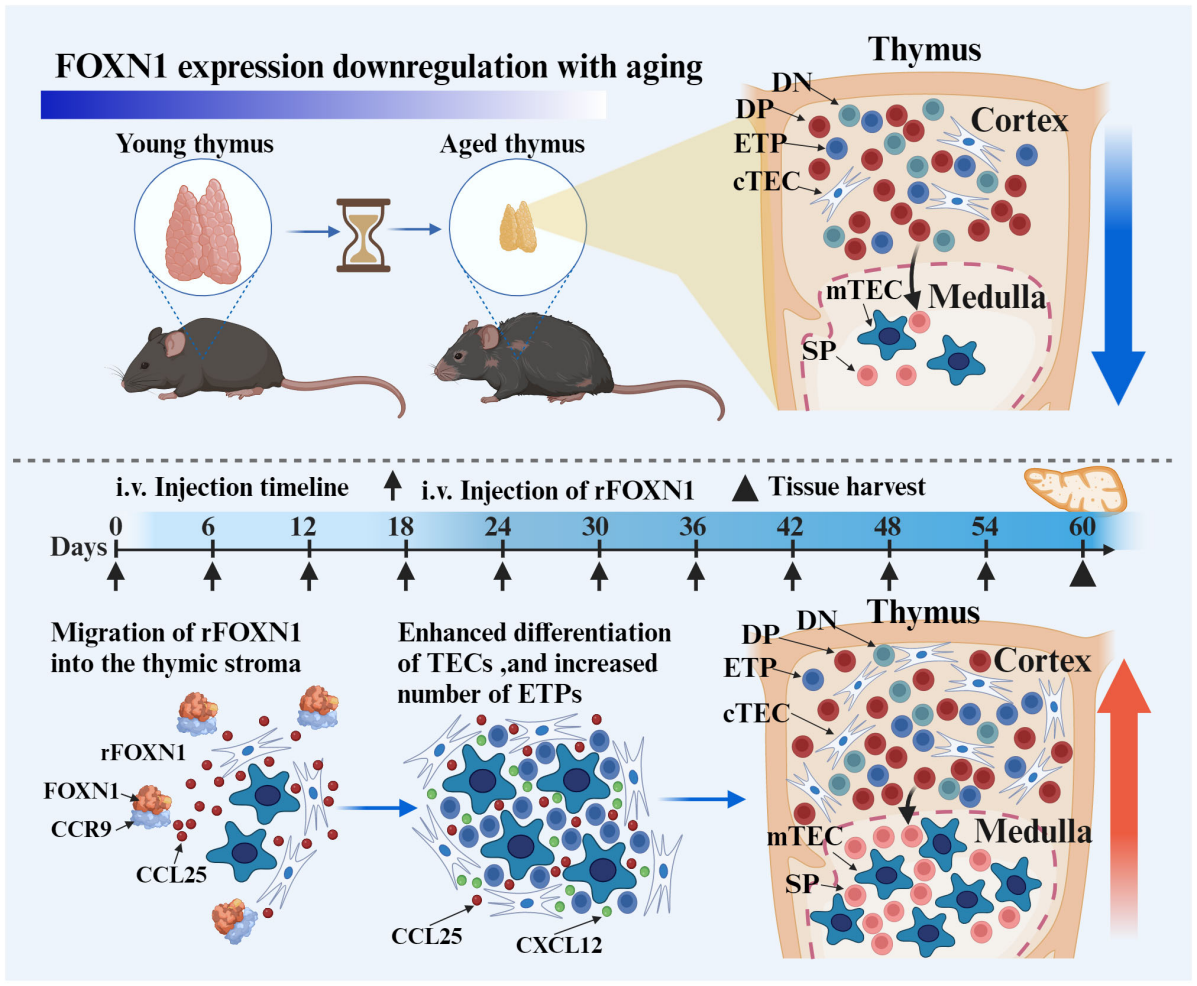
The intravenous (i.v.) injection of rFOXN1 fusion protein, which contains the N-terminal of CCR9, FOXN1 and a protein transduction domain. The rFOXN1 fusion protein enters the thymus, and increases the number of TECs, leading to enhanced thymopoiesis in mice. Created with BioRender.com.

In this study, we develop a rFOXN1 fusion protein that contains the N-terminal of CCR9, human FOXN1 and TAT (CCR9-FOXN1-TAT, simplified as “rFOXN1 fusion protein” also). We show here that, when injected intravenously (i.v.) into 14-month-old mice, the rFOXN1 fusion protein can enter the thymus and enhance T cell generation in the thymus, resulting in an increased number of peripheral T cells (**Scheme 1**). Our results suggest that the rFOXN1 fusion protein has the potential to be used in preventing and treating T cell immunodeficiency in older adults.

## 2 Materials and methods

### 2.1 Mice

C57BL/6 mice were obtained or purchased from NIA and Jackson Laboratory. The mice were used in accordance with protocols approved by the Institutional Animal Care and Use Committee of the University of Connecticut.

### 2.2 Plasmid construction

The extracellular domain of the murine *CCR9* cDNA was amplified using the sense primer (primer 1) containing sequences homologous to the pET-45b (+) vector, and antisense primer (primer 2) containing a linker encoding (Gly4Ser)2 (**Table 1**). The human *FOXN1-TAT* gene was amplified using the sense primer (primer 3) containing the linker and antisense primer (primer 4) containing TAT and 19 bases that are homologous to the pET-45b (+) vector (**Table 1**). The polymerase chain reaction (PCR) products of *CCR9* and *FOXN1-TAT* were combined and subjected to an overlap extension PCR with primers 1 and 4. The entire DNA fragment of the *CCR9-FOXN1-TAT* was cloned into the expression vector pET-45b (+) (Novagen, Gibbstown, NJ) using the In-Fusion Snap Assembly Kit (Clontech, Mountain View, CA) according to the manufacturer’s instructions. To generate control CCR9-MyoD-TAT fusion protein, the *CCR9-MyoD-TAT* gene was similarly cloned into the pET-45b (+) vector. The plasmid constructs were verified by DNA sequencing.

**Table 1:** Primers for *CCR9-FOXN1-TAT* pET-45b (+) construction.

	Sequence 5′–3′
Primer 1	**CCATCACGTGGGTACCGGT**cccacagaactcacaagcct
Primer 2	CGACCCACCACCGCCCGAGCCACCGCCTCCGCTTGC AAACTGCCTGACATT
Primer 3	GGAGGCGGTGGCTCGGGCGGTGGTGGGTCGGTGTC GCTACCCCCGCCGCAGTCT
Primer 4	**tttctttaccagactcgag**CTATCAACGTCTACGTTGCCTTCG

Bold letters indicate the homologous to the pET-45b (+) vector.

### 2.3 Recombinant protein purification and verification

The expression vector containing the *CCR9-FOXN1-TAT* or *CCR9-MyoD-TAT* gene was transformed into BL21(DE3) pLysS bacteria (Novagen). The bacteria were expanded in LB medium with ampicillin until OD_600_ reached 1.0–1.2. Protein expression was induced with 0.4 mM isopropyl-β-D-1-thiogalactopyranoside (IPTG, Novagen) at 37 ° for 4 h. Protein expression level and solubility were determined by the PopCulture quick screen with PopCulture^®^ Reagent (Millipore, USA). Both rFOXN1 and rMyoD fusion proteins were expressed in the inclusion bodies of the bacteria. A freeze/thaw of the cell pellet was prepared by harvesting cells from culture, centrifuging at 10,000 g for 10 min, washing with 50mM Tris-HCl buffer (pH8.0) twice, then freezing the pellet completely at –70°C overnight. Next day, the inclusion bodies from the cell pellet were isolated by the BugBuster^®^ Reagent (Millipore, USA) treatment. The proteins in the inclusion bodies were dissolved in the Inclusion Body Solubilization Reagent (Thermo Scientific). The proteins were further purified by the His Mag Sepharose Ni (Cytiva, USA), and then refolded by a Protein Refolding Kit (Thermo Scientific). The purified proteins were verified by SDS-PAGE, Coomassie Staining and Western blot, and quantified using the Pierce™ BCA Protein Assay Kit (Pierce, Rockford, IL) according to the manufacturer’s instructions.

### 2.4 SDS-PAGE and western blot

Cytoplasmic and nuclear proteins from cells were separated using the NE-PER^®^ Nuclear and Cytoplasmic Extraction Reagents (Thermo Scientific, Rockford, IL). Purified CCR9-FOXN1-TAT and CCR9-MyoD-TAT cytoplasmic or nuclear proteins were loaded on a 10% SDS-PAGE, transferred to a polyvinylidene fluoride membrane, and then incubated with rabbit anti-FOXN1 (Thermo Scientific, or Fitzgerald, Acton, MA), or rabbit anti-MyoD (Santa Cruz Biotechnology, Inc., Dallas, TX), followed by HRP conjugated secondary antibody. The Western Blot was developed with Super Signal^®^ West Pico chemiluminescent Substrate (Thermo Scientific).

### 2.5 rFOXN1 fusion protein and CCL25 binding assay

CCR9-FOXN1-TAT and CCR9-MyoD-TAT proteins were biotinylated by EZ-LinkTM Sulfo-NHS-LC-Biotin kit (Thermo Scientific). FOXN1-TAT and MyoD-TAT proteins that do not contain the CCR9 domain were also biotinylated and used as controls. The biotinylated proteins were incubated with 1 µg/ml CCL25 protein (R&D Systems, Minneapolis, MN) for 1 hour, added to streptavidin beads (Thermo Scientific) and incubated for additional 1 hour. After washing, the proteins were eluted from the beads by adding sample buffer under a reducing condition and subjected to Western blot with anti-FOXN1, MyoD and CCL25 antibodies.

### 2.6 Immunofluorescence

Immunofluorescence analysis of thymus tissues was performed as described (31). Briefly, tissues were incubated in 4% paraformaldeyde for 4 hours followed by incubation in 30% sucrose solution overnight. The tissues were embedded in OCT medium, snap frozen, and subsequently cut into 5 micrometer sections. The sections were stained with Alexa Fluor^®^ 546-conjugated anti-FOXN1 Antibody (Santa Cruz Biotechnology) and rat anti-mouse EpCAM1 antibody (Biolegend), or rat anti-mouse K8 monoclonal antibody (Troma I mAb, raised by P. Brulet and R. Kemler and obtained from the Developmental Studies Hybridoma Bank, University of Iowa, IA), or rabbit anti-mouse K5 polyclonal antibody (Covance Research Products, Denver, PA), followed by AlexaFluor-488-conjugated goat anti-rat or rabbit IgG (Invitrogen). For cultured ESCs or CD45^-^EpCAM1^+^ TECs, the cells were washed and prepared by cytospin, fixed by 5% paraformaldehyde, and stained with the Alexa Fluor^®^ 546-conjugated anti-FOXN1 antibody followed by DAPI nuclear staining. The cells were observed under a Nikon A1R Spectral Confocal microscope (Nikon, Kanagawa, Japan) or Keyence microscope (KEYENCE, USA).

### 2.7 TEC isolation

The thymus was incubated in 0.01 (w/v) liberase (Roche, Nutley NJ) and 0.02% (w/v) DNAse I (Roche) at 37 ° with regular and gentle agitation as described (32). CD45^-^ cells from the single cell suspension of the thymus were negatively selected by CD45 microbeads, and EpCAM1^+^ cells were then positively selected by EpCAM1 microbeads (Miltenyi Biotec, San Diego).

### 2.8 Flow cytometry

Single-cell suspensions of thymocytes and spleen cells were stained with different combinations of the following fluorochrome-conjugated antibodies: CD4, CD8, CD3, CD25, CD44, CD62L, CD117, CD127, EpCAM1, Ly51, CD45, FoxP3, and I-A^b^ (BioLegend, BD Biosciences, San Jose, CA, or eBioscience, San Diego, CA). ETPs were identified as lin^−^IL-7Rα (CD127)^−^c-kit (CD117)^+^CD44^+^CD25^−^thymus cells; a cocktail of antibodies against TER-119, B220, CD19, IgM, Gr-1, CD11b, CD11c, NK1.1, TCRβ, CD3e, and CD8α was used to identify the lin^-^ cells. For Ki67 staining, cells were stained with antibodies for TEC cell surface marker, and then permeabilized with a Cytofix/Cytoperm solution at 4° (BD Biosciences, San Jose, CA). The cells were then stained with FITC-conjugated anti-Ki67 antibody (BioLegend). The cells were analyzed on a LSR II flow cytometer (BD Biosciences); data analysis was done using FlowJo software (Ashland, OR).

### 2.9 TUNEL assay

Cells were stained with antibodies for TEC cell surface markers and with an *in-situ* cell death detection kit (Roche Applied Science). A negative control without the terminal deoxynucleotidyl transferase was also used. The cells were analyzed by flow cytometry.

### 2.10 Real-time qualitative RT-PCR

Total RNA from cells was isolated from tissues, and cDNA was synthesized as described (33). Equal amounts of cDNAs were used for RT-PCR. qRT-PCRs were performed with the Power SYBR green mastermix (Applied Biosystems, UK) using the 7500 real-time PCR system (Applied Biosystems, UK) (34). After normalization to *GAPDH*, samples were plotted relative to control protein-treated group. Primers are summarized in **Supplementary Table 1**.

### 2.11 Statistical analysis

P-values for two groups were based on the two-sided Student’s t test. For comparing means of multiple groups, significance was determined using one‐way ANOVA with Dunnett test, or two-way ANOVA with Tukey test by using SPSS29 software (IBM Corp., Armonk, NY). A confidence level above 95% (*p*<0.05) was determined to be significant.

## 3 Results

### 3.1 Expression, purification, and characterization of rFOXN1 and control fusion proteins

**Figure 1:**
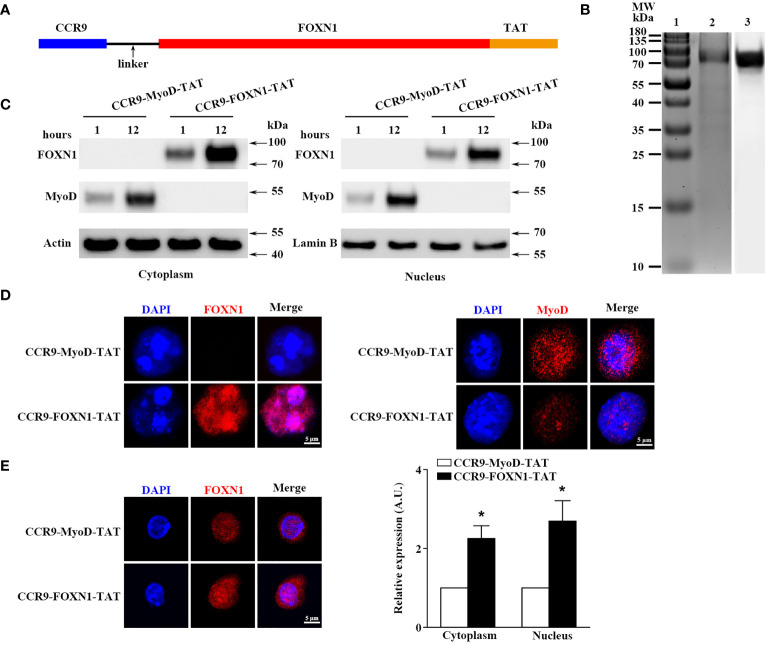
Characterization of the purified rFOXN1 fusion protein. **(A)** A map for the CCR9-FOXN1-TAT fusion protein. **(B)** Gel and blot show purified CCR9-FOXN1-TAT protein; Lane 1: molecular weight markers; 2: Coomassie blue-stained SDS-PAGE; 3: Western blot with anti-FOXN1 antibody. **(C, D)** ESCs from NU/J nude mice were incubated with CCR9-FOXN1-TAT or CCR9-MyoD-TAT protein. **(C)** Western blot analysis of FOXN1 or MyoD protein from cytoplasmic and nuclear extractions. Actin and Lamin B were used as loading controls for cytoplasmic and nuclear proteins, respectively. **(D)** Immunofluorescence analysis of CCR9-FOXN1-TAT or CCR9-MyoD-TAT protein in the cytoplasm and nucleus after 12-hour incubation. **(E)** CD45^-^EpCAM1^+^ primary TECs were isolated from B6 mice and incubated with CCR9-FOXN1-TAT or CCR9-MyoD-TAT protein. Immunofluorescence analysis of FOXN1 protein in the cytoplasm and nucleus after 12-hour incubation. (left) Representative images are shown and (right) the FOXN1 levels were quantitatively evaluated and shown as relative expression levels. **(B–E)** Data are representative of three independent experiments with similar results. **(E)** Groups comparisons were carried out by two-way ANOVA (protein × location). A.U. means Arbitrary Units. * *p*<0.05 compared with control protein-treated mice.

To produce rFOXN1 fusion protein, the extracellular domain of the murine *CCR9* and human *FOXN1* genes were connected by a flexible linker encoding (Gly4Ser)_2._ The *TAT* DNA was added to the C-terminal of the *FOXN1* (**Figure 1A**). The entire DNA fragment of the *CCR9-FOXN1-TAT* was cloned into a prokaryotic expression vector pET-45b (+) which was then transformed into Rosetta 2 (DE3) bacteria. The CCR9-FOXN1-TAT fusion protein was purified from the bacteria. A relatively high purity of rFOXN1 fusion protein was obtained, as determined by Coomassie blue-stained SDS-PAGE (**Figure 1B**, lane 2). The identity of the protein was verified by Western blot using an anti-human FOXN1 antibody (**Figure 1B**, lane 3). For controls, a human MyoD fusion protein that contained murine CCR9 and human MyoD-TAT was similarly cloned, expressed and purified with the same system; the purified CCR9-MyoD-TAT protein was verified by SDS-PAGE and Western blot using an anti-human MyoD antibody (data not shown).

We then determined whether the rFOXN1 fusion protein, when added into cultured cells, could translocate into the cytoplasm and nucleus of cultured cells. Mouse embryonic stem cells (ESCs) from NU/J nude mice that lack FOXN1 protein were cultured in the presence of rFOXN1 or control rMyoD fusion protein. One or 12 hours later, nuclear and cytoplasmic protein extracts were prepared and subjected to Western blot analysis using the anti-FOXN1 or MyoD antibody (26). The FOXN1 or MyoD fusion protein was present in both the cytoplasm and nucleus after 1 and 12 hours, but greater in nucleus after the longer incubation (**Figure 1C**; **Supplementary Figure 1**). The results were confirmed by immunofluorescence staining showing that the rFOXN1 or MyoD fusion protein was present in both the cytoplasm and nucleus, with more protein in the nucleus than in the cytoplasm after 12-hour incubation (**Figure 1D**). Furthermore, we isolated CD45^-^EpCAM1^+^ primary TECs from C57BL/6 (B6) mice and incubated them with the rFOXN1 fusion protein or control protein. Since the anti-FOXN1 antibody reacts with both the endogenous mouse FOXN1 and the human rFOXN1 fusion protein, we detected the total expression levels of both FOXN1 proteins with immunofluorescence. As shown in **Figure 1E**, FOXN1 protein levels in both the cytoplasm and nucleus were significantly increased after the rFOXN1 fusion protein was added in the cultures. Collectively, the results suggest that the rFOXN1 fusion protein can translocate from the cell surface into the cytoplasm and nucleus.

### 3.2 The rFOXN1 fusion protein can enter the thymus when injected peripherally

To determine the ability of the rFOXN1 or rMyoD fusion protein to bind to the CCR9 ligand, CCL25, CCR9-FOXN1-TAT and CCR9-MyoD-TAT, as well as control FOXN1-TAT and MyoD-TAT proteins were labelled with biotin. The biotinylated proteins were incubated with CCL25 protein and then added to streptavidin beads. After washing, the proteins were eluted from the beads by adding sample buffer under a reducing condition and subjected to Western blot with anti-FOXN1, MyoD and CCL25 antibodies. We detected both FOXN1 or MyoD, and CCL25 from CCR9-FOXN1-TAT or CCR9-MyoD-TAT elution, but only FOXN1 or MyoD from FOXN1-TAT or CCR9-MyoD elution (**Supplementary Figure 2**). The results suggest that CCR9-FOXN1-TAT and CCR9-MyoD-TAT can bind to CCL25.

**Figure 2:**
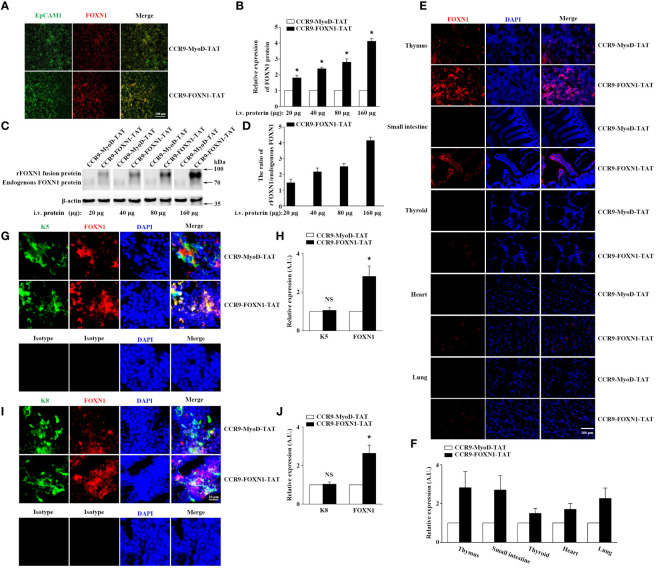
rFOXN1 fusion protein enters the thymus when injected peripherally. B6 mice were injected i.v. with graded doses of CCR9-FOXN1-TAT or CCR9-MyoD-TAT (20, 40, 80, and 160 µg). One day later, the thymi were harvested and analyzed for FOXN1 expression levels by **(A, B)** immunofluorescence and **(C, D)** Western blot using anti-FOXN1 antibody. **(A)** Representative images of EpCAM1^+^ TECs (green color) and the expression of FOXN1 (red color). **(B)** Relative expression levels of FOXN1 in the thymus of CCR9-FOXN1-TAT or CCR9-MyoD-TAT-treated mice. The expression levels of FOXN1 in the control protein-treated mice were defined as 1. **(C)** Representative images of the expression of endogenous mouse FOXN1 and human rFOXN1 fusion protein. **(D)** The ratio of human rFOXN1 fusion protein/endogenous mouse FOXN1 protein in CCR9-FOXN1-TAT-treated mice. B6 mice were injected i.v. with 80 µg CCR9-FOXN1-TAT or CCR9-MyoD-TAT. One day later, the thymus, small intestine, thyroid, heart, and lung were harvested and analyzed for FOXN1 (red color) expression levels **(E–J)** by immunofluorescence using anti-FOXN1 antibody, and the thymic sections were also analyzed for K5 (green color) expression levels **(G, H)**, or K8 (green color) expression levels **(I, J)** by immunofluorescence using anti-K8 or anti-K5 or isotype antibodies, sections **(E, G, I)** were stained with DAPI for nuclear staining. **(B, D, F, H, J)** Data are shown as mean ± SD (n = 8 mice/group) and are representative of three independent experiments with similar results. Overall groups comparisons **(D)** were carried out by one-way ANOVA with Dunnett test, groups comparisons for B, F, H, and J were carried out by two-way ANOVA with *post-hoc* test. A.U. means Arbitrary Units. * *p*<0.05 compared with control protein-treated mice.

To determine the ability of the rFOXN1 fusion protein to travel to the thymus from the periphery, B6 mice were injected i.v. with graded doses of CCR9-FOXN1-TAT or control CCR9-MyoD-TAT fusion protein (20, 40, 80, and 160 µg). One day later, the thymic sections were stained with antibodies against FOXN1 and EpCAM1 (to identify total TECs). As shown in **Figures 2A, B**, i.v. injection of the rFOXN1 fusion protein resulted in increased levels of FOXN1 in the TECs in a dose-dependent manner with more than 2-, 2.5- and 4-fold increase with 40, 80 and 160 µg rFOXN1 fusion protein, as compared with respective doses of control protein. We then performed Western blot to confirm the levels of FOXN1. The lysis of the thymus tissues was analyzed by Western blot using the anti-FOXN1 antibody that also reacted with both mouse and human FOXN1. Since the molecular weight of the rFOXN1 fusion protein is higher than that of endogenous mouse FOXN1, we could separate the expression levels of the two FOXN1 proteins with this method. As shown in **Figures 2C, D**, the expression levels of the endogenous mouse FOXN1 protein were not significantly changed after injection of graded doses of the FOXN1 or control fusion protein. In contrast, the levels of the rFOXN1 fusion protein in the thymus were significantly increased in a dose-dependent manner after i.v. injection of the rFOXN1 fusion protein (**Figures 2C, D**; **Supplementary Figure 1**). Similar trends were also observed when CD45^-^EpCAM1^+^ TECs were purified from the thymus, and the cytoplasmic and nuclear fractions in the TECs were separated and analyzed for FOXN1 protein level by Western blot (data not shown).

To determine the potential of the rFOXN1 fusion protein to enter other organs, B6 mice were injected i.v. with 80 µg CCR9-FOXN1-TAT or CCR9-MyoD-TAT fusion protein. One day later, the thymus and other organs including the small intestine, thyroid, heart, and lung were harvested, and frozen sections of the organs were also prepared and analyzed for the expression of FOXN1 by immunofluorescence. We detected a significant increase of FOXN1 protein in the thymus and the small intestine after CCR9-FOXN1-TAT, but not control CCR9-MyoD-TAT, FOXN1-TAT, or MyoD-TAT protein injected i.v. (**Figures 2E, F**; **Supplementary Figure 3**). Only a few of CCR9-FOXN1-TAT fusion protein entered other organs (**Figures 2E, F**). The addition of DAPI nuclear staining showed an overlap of signals from rFOXN1 and nuclear dye in thymus (**Figure 2E**), suggesting the rFOXN1 fusion protein went to the nucleus of the cells. By using K5 to identify medullary TECs (mTECs) or keratin 8 (K8) to identify cortical TECs (cTECs), we found that CCR9-FOXN1-TAT could enter both mTECs and cTECs (**Figures 2G–J**). In contrast, little or none of the CCR9-FOXN1-TAT fusion protein entered thymocytes, endothelial cells and macrophages although some of the protein entered fibroblasts (**Supplementary Figure 4**). Taken together, the results suggest that the rFOXN1 fusion protein can transport to the thymus, and preferentially enter TECs when injected peripherally.

### 3.3 The rFOXN1 fusion protein increases the number of total TECs and TEC subsets in old mice

**Figure 3:**
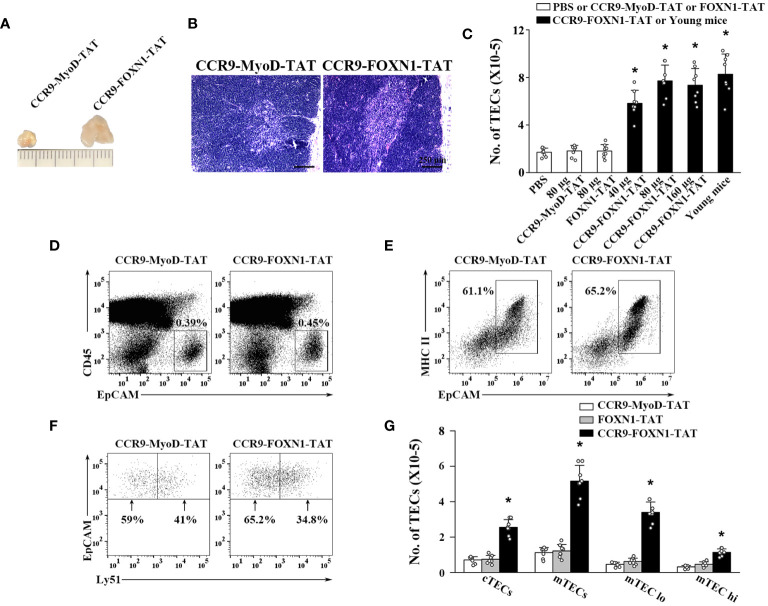
rFOXN1 fusion protein increases the number of total TECs and TEC subsets in old mice. B6 mice (14-month-old) were injected i.v. with graded doses of CCR9-FOXN1-TAT (40, 80, and 160 µg), control CCR9-MyoD-TAT or FOXN1-TAT protein (80 µg), or PBS at 6 day-intervals. Two months later, the thymi were harvested and analyzed by flow cytometry. **(A)** Representative images of 80 µg CCR9-FOXN1-TAT or CCR9-MyoD-TAT protein-treated thymus. **(B)** The thymic architecture was analyzed by H&E staining. **(C)** The number of total TECs (CD45^-^EpCAM^+^), and **(D–G)** the percentages and numbers of cTECs (CD45^-^EpCAM^+^MHC II^+^Ly51^+^), mTECs (CD45^-^EpCAM^+^MHC II^+^Ly51^-^), mTECs^lo^ and mTECs^hi^ TEC subsets from the 80 µg CCR9-FOXN1-TAT or CCR9-MyoD-TAT protein treated mice are shown. **(C)** The number of total TECs in untreated 1-month-old mice was also used as a control. Representative flow cytometric profiles showing the percentage of **(D)** CD45^-^ thymic stromal cells, **(E)** EpCAM^+^MHC II^+^ TECs in CD45^-^ thymic stromal cells and **(F)** Ly51^+^ cTEC and Ly51^-^ mTECs in CD45^-^EpCAM^+^MHC II^+^ TECs. **(C, G)** Data are shown as mean ± SD (n = 8 mice/group) and are representative of three independent experiments with similar results. Statistical significance was determined by **(C)** one‐way ANOVA with Dunnett test and **(G)** two-way ANOVA with *post-hoc* test. * *p*<0.05 compared with control rMyoD protein-treated mice.

We then determined the ability of the rFOXN1 fusion protein to increase the number of TECs in old mice. Fourteen-month-old B6 mice were injected i.v. with graded doses of CCR9-FOXN1-TAT, control CCR9-MyoD-TAT or FOXN1-TAT protein (40, 80, and 160 µg) at 6 day-intervals. PBS was also used as a negative control. Two months later, the thymi were harvested. As shown in **Figure 3A**, CCR9-FOXN1-TAT-treated thymus was significantly larger than control CCR9-MyoD-TAT or FOXN1-TAT protein-treated thymus. We analyzed the thymic architecture by H&E staining. The control protein-treated thymus had a disorganized compartmentalization of cortical and medullary areas, and a reduction in medullary area (**Figure 3B**). In contrast, CCR9-FOXN1-TAT protein-treated thymus displayed a distinctive cortical and medullary area and increased medulla (**Figure 3B**), a structure similar to the young thymus.

We then analyzed the number of total TECs (CD45^-^EpCAM^+^) by flow cytometry. Control CCR9-MyoD-TAT or FOXN1-TAT protein did not affect the number of total TECs at any dose, as compared to PBS treatment (**Figure 3C** and data not shown). In contrast, the numbers of total TECs in 40, 80, and 160 µg CCR9-FOXN1-TAT protein-treated mice were increased 3-, 4-, and 3.9-fold, respectively, above those in control CCR9-MyoD-TAT protein-treated mice (**Figure 3C**). The number of total TECs in 80 µg rFOXN1 fusion protein-treated old mice almost reached that in 1-month-old untreated young mice (**Figure 3C**).

TECs can be broadly divided into cTECs and mTECs, which can be identified using flow cytometric analysis with anti-Ly51 and anti-UEA antibodies (Ly51^+^UEA^-^ for cTECs and Ly51^-^UEA^+^ for mTECs). CCR9-FOXN1-TAT protein increased the numbers of both CD45^-^EpCAM^+^MHC II^+^Ly51^+^ cTECs and CD45^-^EpCAM^+^MHC II^+^Ly51^-^ mTECs with a greater effect on mTECs, as compared with the rMyoD control protein (**Figures 3D–G**). Similar results were obtained when CD45^-^EpCAM^+^MHC II^+^UEA^-^ cTECs and CD45^-^EpCAM^+^MHC II^+^UEA^+^ mTECs were analyzed (data not shown). mTECs can be further divided into mTECs^lo^ and mTECs^hi^ subsets based on the expression level of MHC II, and the former are considered as immature mTECs (35–37). As shown in **Figure 3G**, CCR9-FOXN1-TAT protein increased the number of both mTECs^lo^ and mTECs^hi^. Taken together, our results suggest that the rFOXN1 fusion protein treatment increases the number of total TECs and TEC subsets in old mice.

To determine whether the increased number of TECs by the rFOXN1 fusion protein was due to enhanced cell survival and/or proliferation, we first analyzed cell survival using a TUNEL apoptosis detection assay. The percentages of TUNEL^+^ apoptotic cells in cTECs and mTECs were reduced by 64% and 62%, respectively, in CCR9-FOXN1-TAT protein-treated mice as compared to the control protein-treated mice (**Supplementary Figure 5A**). We then analyzed TEC proliferation by measuring Ki67^+^ cells via flow cytometry. CCR9-FOXN1-TAT protein did not significantly increase the percentage of Ki67^+^ cells in cTECs but increased the percentage of Ki67^+^ cells in mTECs 1.7-fold over control protein (**Supplementary Figure 5B**). Collectively, our data suggest that the rFOXN1 fusion protein increased the number of TECs by enhancing the survival of both cTECs and mTECs, as well as the proliferation of mTECs.

### 3.4 The rFOXN1 fusion protein increases the number of total thymocytes and thymocyte subsets including early thymic progenitors in old mice

**Figure 4:**
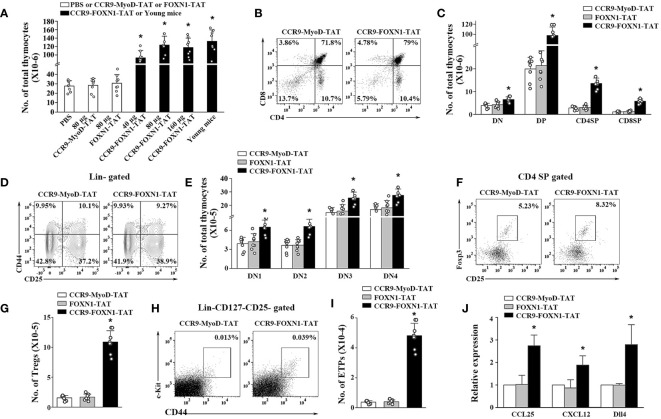
The rFOXN1 fusion protein increases the number of total thymocytes and thymocyte subsets in old mice. B6 mice (14-month-old) were injected i.v. with CCR9-FOXN1-TAT, control CCR9-MyoD-TAT or FOXN1-TAT protein at 6 day-intervals as in **Figure 3**. Two months later, the thymi were harvested and analyzed by flow cytometry. **(A)** The number of CD45^+^ total thymocytes from all groups of mice including a group of untreated 1-month-old mice. **(B–I)** The percentages and numbers of thymocyte subsets from the 80 µg CCR9-FOXN1-TAT or control protein treated mice. **(B)** Representative flow cytometric profiles showing the percentage of CD4 and CD8 DN, DP, and SP thymocytes (analyzing 10,000 cells per sample). **(C)** Number of CD4 and CD8 DP, CD4 SP and CD8 SP thymocytes. **(D)** Representative flow cytometric profiles showing the percentage of DN1-DN4 thymocytes after gating on lin^-^ cells (one million cells per sample). **(E)** Number of DN1-DN4 thymocytes. **(F)** Representative flow cytometric profiles showing the percentage of CD4^+^CD25^+^FoxP3^+^ Treg cells. **(G)** Number of the Treg cells. **(H)** Representative flow cytometric profiles showing the percentage of lin^-^c-kit^+^IL-7Rα^-^CD44^+^CD25^-^ ETPs in total thymocytes (one million cells per sample). **(I)** Number of ETPs. **(J)** CD45^-^ thymic stromal cells were isolated from the 80 µg CCR9-FOXN1-TAT or control protein-treated old mice, and analyzed for the expression levels of CCL25, CXCL12, and Dll4 by qRT-PCR. The expression levels of the genes in control MyoD protein-treated mice are defined as 1. **(A, C, E, G, I, J)** Data are shown as mean ± SD (n = 6–8 mice/group) and are representative of three independent experiments with similar results. Statistical significance was determined by **(A, G, I)** one‐way ANOVA with Dunnett test and **(C, E, J)** two-way ANOVA with *post-hoc* test. * *p*<0.05 compared with control protein-treated mice.

We have previously shown that the increased number of TECs in the murine rFOXN1 protein treated mice that have undergone hematopoietic stem cell transplantation subsequently increased the number of thymocytes (26). We therefore determined whether CCR9-FOXN1-TAT protein has a similar effect on old mice. Like the effect on TECs, a parallel dose-dependent increase in the number of CD45^+^ total thymocytes was observed; the numbers of total thymocytes in 40, 80, and 160 µg CCR9-FOXN1-TAT fusion protein-treated mice were increased 3.3-, 4.3-, and 4.1-fold, respectively, above those in control CCR9-MyoD-TAT or control FOXN1-TAT-treated mice (**Figure 4A**). Again, the number of total thymocytes in 80 µg CCR9-FOXN1-TAT protein-treated old mice almost reached that in untreated 1-month-old young mice (**Figure 4A**).

Thymocytes can be divided into the following major subsets: CD4 and CD8 double negative (DN), double positive (DP), CD4 single positive (SP), and CD8 SP thymocytes. CCR9-FOXN1-TAT protein decreased the percentages of DN thymocytes but increased the percentages of DP and CD8 SP thymocyte subsets (**Figure 4B**; **Supplementary Figure 6A**). Because CCR9-FOXN1-TAT protein increased the number of total thymocytes, it increased the number in each of the thymocytes subsets including DN thymocytes (**Figure 4C**). DN thymocytes can be further divided into DN1, DN2, DN3 and DN4 subsets based on the expressions of CD44 and CD25. CCR9-FOXN1-TAT did not significantly change the percentage of the subsets but increased the cell numbers of each subset (**Figures 4D, E**; **Supplementary Figure 6B**). Since natural regulatory T cells (Tregs) also develop in the thymus (38), we analyzed these cells. CCR9-FOXN1-TAT protein treatment increased the percentage and number of CD4^+^CD25^+^FoxP3^+^ Tregs in the old mice (**Figures 4F, G**; **Supplementary Figure 6C**).

The early thymic progenitor (ETPs) (lin^-^ c-kit^+^ IL-7Rα^-^ CD44^+^CD25^-^) are considered to represent canonical intrathymic T-cell progenitors (39–41). CCR9-FOXN1-TAT protein treatment increased both the percentage and number of ETPs (**Figures 4H, I**; **Supplementary Figure 6D**). To determine whether the increased ETPs were due to CCR9-FOXN1-TAT induced-enhanced expression of chemokines that can attract T-cell precursors to the thymus, we analyzed the expression levels of CCL25 and CXCL12 that are the transcriptional targets of FOXN1 (24, 42, 43). We found that the expression levels of CCL25 and CXCL12 in CD45^-^ thymic stromal cells were increased after CCR9-FOXN1-TAT protein treatment (**Figure 4J**). Since delta-like (Dll) 4 is also the target of FOXN1 and plays a critical role in T cell development (43, 44), we analyzed Dll4 and found that the expression level of this gene was also increased after CCR9-FOXN1-TAT treatment (**Figure 4J**). Taken together, our data suggests that the rFOXN1 fusion protein treatment increases the number in all thymocyte subsets including ETPs, which is related to increased expression of CCL25, CXCL12 and Dll4 in thymic stromal cells.

### 3.5 The rFOXN1 fusion protein-treated aged mice have increased the number of peripheral T cells

**Figure 5:**
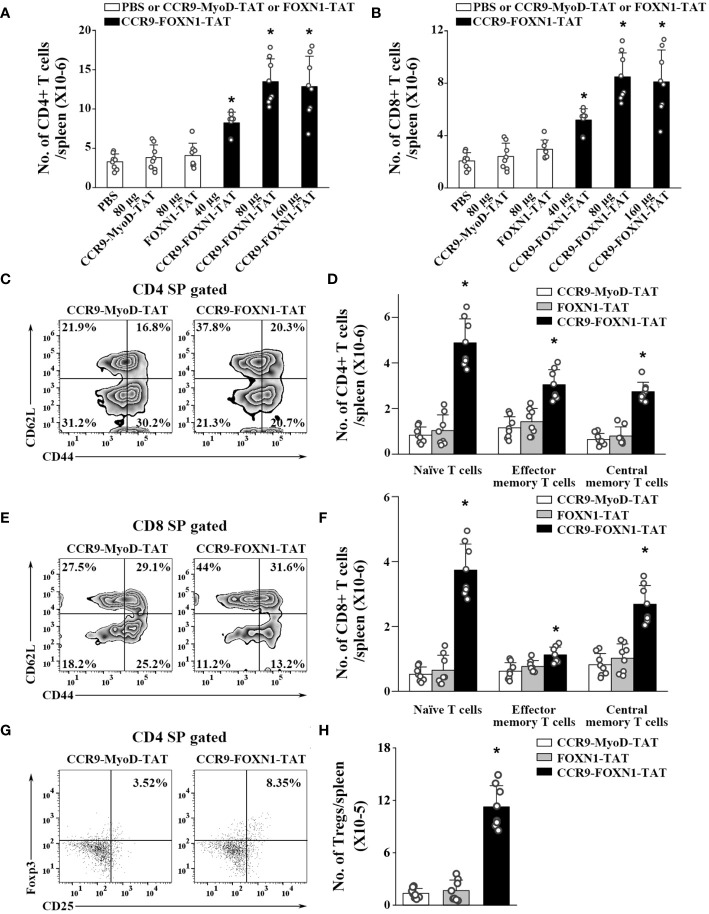
The rFOXN1 fusion protein-treated old mice have increased number of peripheral T cells. B6 mice (14-month-old) were injected i.v. with CCR9-FOXN1-TAT, control CCR9-MyoD-TAT or FOXN1-TAT at 6 day-intervals as in **Figure 4**. Two months later, the spleens were harvested and analyzed by flow cytometry. **(A, B)** The number of total **(A)** CD4^+^ and **(B)** CD8^+^ T cells from all groups of mice. **(C–H)** The percentages and numbers of CD4^+^ and CD8^+^ T cell subsets from the 80 µg CCR9-FOXN1-TAT, or control protein treated mice. **(C, E)** Representative flow cytometric profiles showing the percentage of CD4 and CD8 CD44^lo^CD62L^hi^ naïve, CD44^hi^CD62L^lo^ effector memory, and CD44^hi^CD62L^hi^ central memory T cells. **(D, F)** The number of CD4 and CD8 naïve, effector memory, and central memory T cells. **(G)** Representative flow cytometric profiles showing the percentage of CD4^+^CD25^+^FoxP3^+^ Tregs. **(H)** The number of the Tregs. **(A, B, D, F, H)** The data are shown as mean ± SD (n = 8 mice/group) and are representative of three independent experiments with similar results. Statistical significance was determined by **(A, B, H)** one‐way ANOVA with Dunnett test and **(D, F)** two-way ANOVA with *post-hoc* test. * *p*<0.05 compared with control protein-treated mice.

We also determined whether the enhanced thymopoiesis in the rFOXN1 fusion protein-treated aged mice resulted in an increased number of peripheral T cells. The spleens were harvested from the CCR9-FOXN1-TAT, control CCR9-MyoD-TAT or FOXN1-TAT-treated old mice 2 months later. CCR9-FOXN1-TAT protein treatment significantly increased the number of total CD4^+^ and CD8^+^ T cells; 40, 80, and 160 µg CCR9-FOXN1-TAT protein increased 2.2-, 3.5-, and 3.4-fold CD4^+^ T cells and CD8^+^ T cells, respectively, above those in control protein-treated mice (**Figures 5A, B**).

T cells can be divided into CD44^lo^CD62L^hi^ naïve, CD44^hi^CD62L^lo^ effector memory, and CD44^hi^CD62L^hi^ central memory T cells. CCR9-FOXN1-TAT protein significantly increased the percentages and numbers of CD4^+^ and CD8^+^ naïve T cells (**Figures 5C–F**; **Supplementary Figure 7**). Although CCR9-FOXN1-TAT protein decreased the percentages of CD4^+^ and CD8^+^ effector memory T cells, because CCR9-FOXN1-TAT increased the number of total CD4^+^ and CD8^+^ T cells, it also increased the numbers of these T cells (**Figures 5C–F**; **Supplementary Figure 7**).

We then analyzed CD4^+^CD25^+^FoxP3^+^ Tregs in the spleen. CCR9-FOXN1-TAT treatment increased the percentage and number of CD4^+^CD25^+^FoxP3^+^ Tregs in the old mice (**Figures 5G**, **6H**; **Supplementary Figure 7**). The increased numbers of CD4 and CD8 T cells, as well as Tregs were also similarly observed in lymph nodes and blood (data not shown). Collectively, our results suggested that the enhanced thymopoiesis in the rFOXN1 fusion protein-treated old mice resulted in an increased number of peripheral T cells.

## 4 Discussion

Given CCR9’s stringent specificity for its ligand CCL25 and the abundant expression of CCL25 in thymic tissue, particularly within the thymic stroma (28–30), we fused the N-terminal of murine CCR9 to human FOXN1. We have shown here that the rFOXN1 fusion protein can enter the thymus, especially TECs, after injected peripherally. The results suggest that CCR9 can direct FOXN1 protein to move to the thymus (with preferentially to TECs) from the blood. Our data are consistent with the report that a fusion protein containing the N-terminal of CCR9 and IL-7, when injected peripherally, increased the content of IL-7 in the thymus, resulting in enhanced thymopoiesis (28). In addition to the thymus, the rFOXN1 fusion protein also enter the small intestine, which is consistent with the report that CCL25 is predominantly expressed by thymic and intestinal epithelial cells (45). We have shown that the rFOXN1 fusion protein preferentially enters TECs and fibroblasts; this probably because these cells express and produce CCL25, leading to higher CCL25 concentrations on the cell surface and surrounding areas. Although this small amount of the rFOXN1 fusion enters the small intestine, we did not observe obvious side effects after the rFOXN1 fusion protein treatment.

We have also shown that i.v. injection of the rFOXN1 fusion protein increased the number of TECs in old mice, which was due to enhanced survival of both cTECs and mTECs, as well as increased proliferation of mTECs. The results are consistent with our data that rFOXN1 fusion protein had more significant effects on mTECs (**Figure 3G**) and other reports that mTECs are more FOXN1-dependent than cTECs (16, 18, 24).

The increased number of TECs in rFOXN1 fusion protein-treated old mice resulted in enhanced T cell generation in the thymus, leading to increased numbers of T cells in the peripheral lymphoid organs. Although the rFOXN1 fusion protein increases the number of TECs and thymocytes in a dose-dependent manner, administration of the protein at a 160 µg does not have greater effect than the 80 µg does. The data are consistent with our own and other reports that the FOXN1 dosage needs to be tightly controlled (16, 18, 19, 21, 24, 26).

In our previous studies, we used murine FOXN1 protein (26). For future potential clinical applications, we generated human FOXN1 fusion protein in this study. We found that the human FOXN1 fusion protein could also affect mouse TECs similarly to murine FOXN1 protein (26), likely because mouse and human FOXN1 proteins have a high homology. It has been reported that 85% of amino acids in human FOXN1 protein are identical to the murine form and both proteins are also identical in length (648 aa) (46).

The rFOXN1 fusion protein-treated old mice had increased number of ETPs in the thymus (**Figure 4I**) and increased expression of chemokines CCL25 and CXCL12 in thymic stromal cells (**Figure 4J**). It has been reported that CCL25 and CXCL12 are the transcriptional targets of FOXN1 (24, 42, 43). It is possible that the increased expression of chemokines results in enhanced migration of T-cell precursors to the thymus. It is also possible that rFOXN1 directly acts on ETPS and/or the precursors for ETPs since it has been reported that not only the number of thymic ETPs, but also that of bone marrow hematopoietic stem cells and multipotent progenitors in aged FOXN1 transgenic mice was higher than in age-matched WT mice (19). The number of Tregs in the rFOXN1 fusion protein-treated old mice was also increased (**Figure 5H**), which was likely due to the increased generation of TECs that support the development of natural Tregs (47, 48). We cannot exclude the possibility that the rFOXN1 fusion protein affects bone marrow hematopoietic stem cells and progenitors. Our studies have limitations because we did not encompass the analysis of the percentage and number of bone marrow hematopoietic stem cells and multipotent progenitors in the bone marrow of the rFOXN1 fusion protein-treated mice.

Age-dependent thymic involution/atrophy can be caused by both TEC and ETP degeneration. rFOXN1 fusion protein treatment increases both TECs and ETPs in old mice. It has been reported that a reduced expression or the deletion of a single gene, *FOXN1*, results in phenotypes similar to age-dependent thymic involution. Furthermore, overexpression of FOXN1 attenuates age-associated thymic involution (19, 21, 24). Our results showing that administration of the rFOXN1 fusion protein increases the number of TECs and thymopoiesis are consistent with these reports, suggesting that FOXN1 plays a critical role in maintaining adult TECs.

## 5 Conclusions

We have shown that administration of the human rFOXN1 fusion protein in old mice can increase the number of TECs, resulting in enhanced T cell generation in the thymus, leading to an increased number of peripheral T cells. Our results suggest that the rFOXN1 fusion protein has the potential to be used in preventing and treating T cell immunodeficiency in older adults.

## Data availability statement

The raw data supporting the conclusions of this article will be made available by the authors, without undue reservation.

## Ethics statement

The animal study was approved by Institutional Animal Care and Use Committee of the University of Connecticut. The study was conducted in accordance with the local legislation and institutional requirements.

## Author contributions

JZ: Data curation, Investigation, Methodology, Validation, Visualization, Writing – original draft, Writing – review & editing. RH: Investigation, Methodology, Writing – review & editing. KL: Investigation, Writing – review & editing. ZZ: Investigation, Writing – review & editing. LL: Formal analysis, Funding acquisition, Supervision, Writing – original draft, Writing – review & editing.
