# Supplementary material for: Recombinant FOXN1 fusion protein increases T cell generation in old mice
Source: Front Immunol. 2024 Jul 12;15:1423488. doi: 10.3389/fimmu.2024.1423488 (PMC11272594; doi:10.3389/fimmu.2024.1423488)
Supplement: Supplementary file 1 [file DataSheet_1.pdf]

Supplementary Material

1 Supplementary Figures

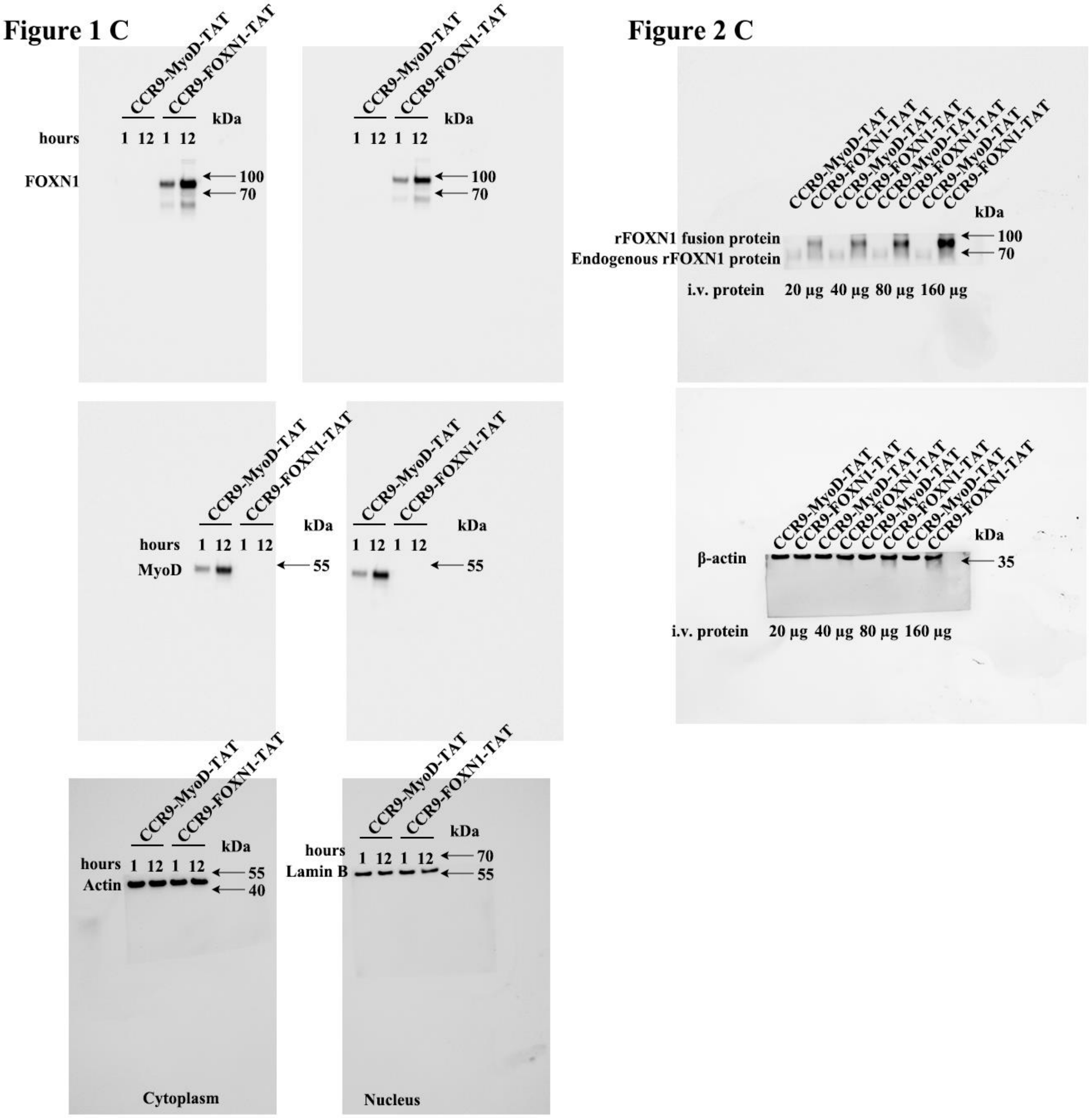

Supplemental Figure 1. Larger Western blot images for Figure 1C and Figure 2C.

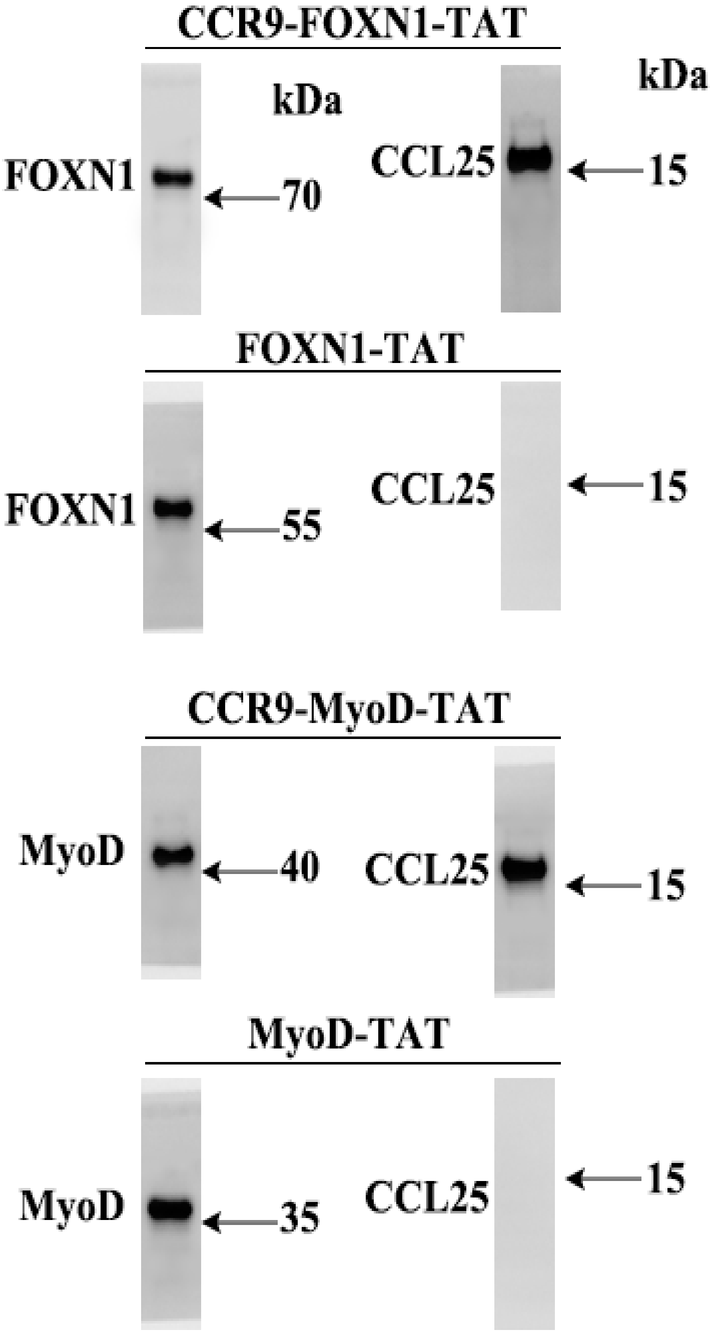

Supplemental Figure 2. rFOXN1 fusion protein can bind to CCL25 protein. CCR9-FOXN1-TAT, CCR9-MyoD-TAT, and control FOXN1-TAT and MyoD-TAT proteins were biotinylated, incubated with CCL25, and then added to streptavidin beads. Sample buffer under a reducing condition was added to the beads and Western blot with anti-FOXN1, MyoD and CCL25 antibodies was performed. Data are representative of three independent experiments with similar results.

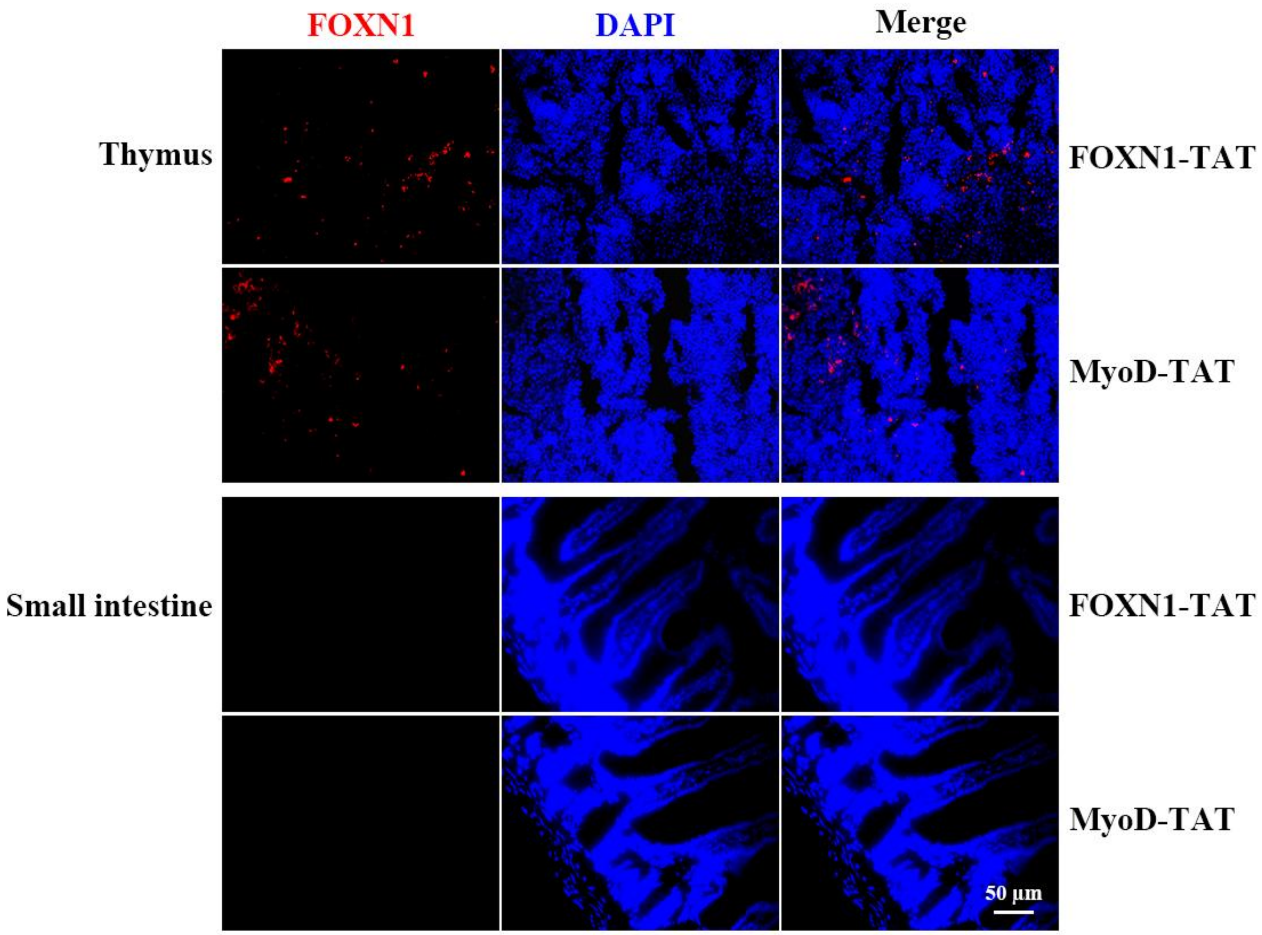

Supplemental Figure 3. Control FOXN1-TAT does not enter the thymus when it is injected i.v. into mice. B6 mice were injected i.v. with 80 µg FOXN1-TAT or MyoD-TAT protein. One day later, the thymus and small intestine were harvested and analyzed for FOXN1 (red color) expression levels by immunofluorescence using anti-FOXN1 antibody. Data are representative of three independent experiments with similar results.

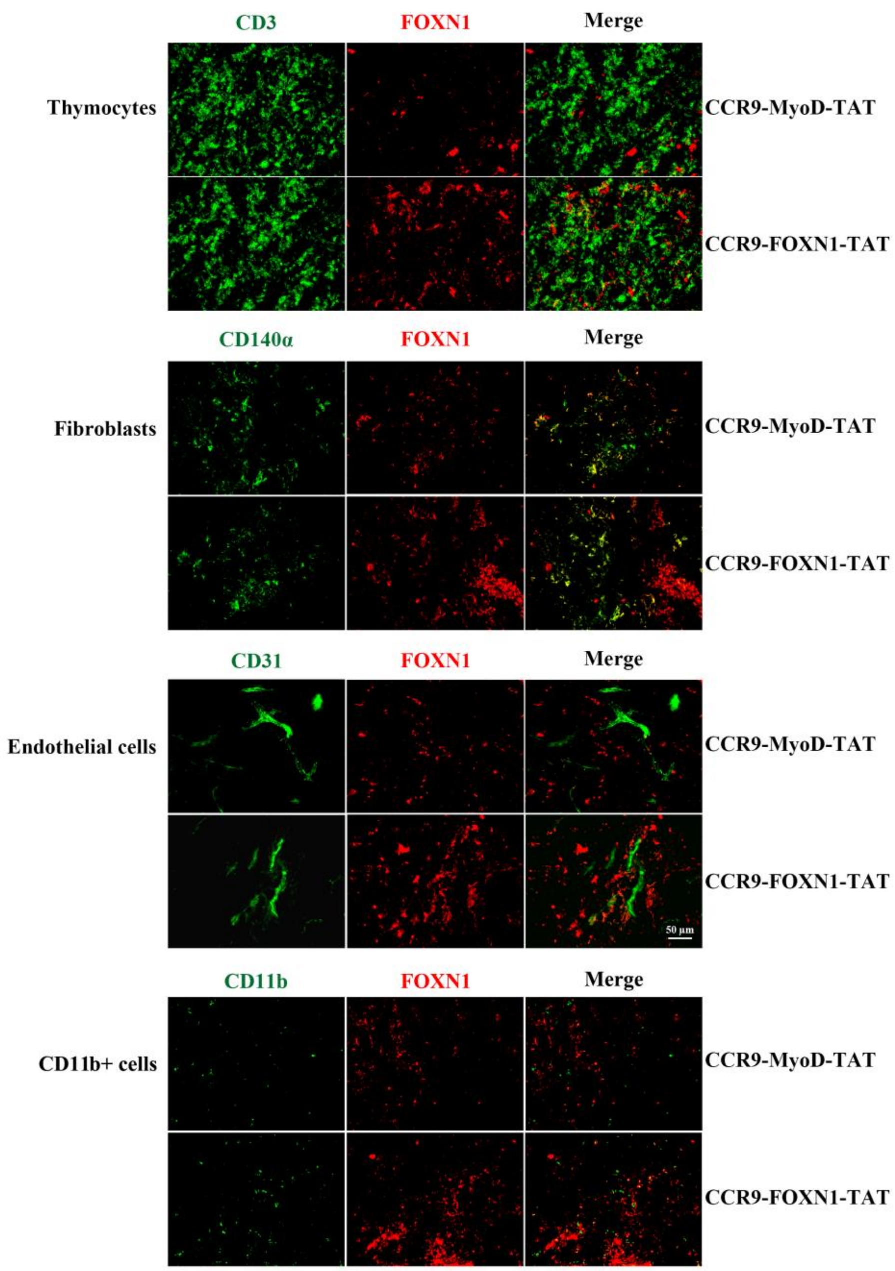

Supplemental Figure 4. Little or none of CCR9-FOXN1-TAT fusion protein enters thymocytes and endothelial cells when it is injected i.v. into mice. B6 mice were injected i.v. with 80 µg CCR9-FOXN1-TAT or CCR9-MyoD-TAT protein. One day later, the sections of the thymus were stained with anti-FOXN1, and anti-CD3, CD31, CD140α, or CD11b antibody. The expression of FOXN1 (red color) in $CD3^+$ thymocytes, $CD31^+$ endothelial cells, CD140α^+ fibroblasts, and $CD11b^+$ macrophages (green color) was analyzed by immunofluorescence. Data are representative of three independent experiments with similar results.

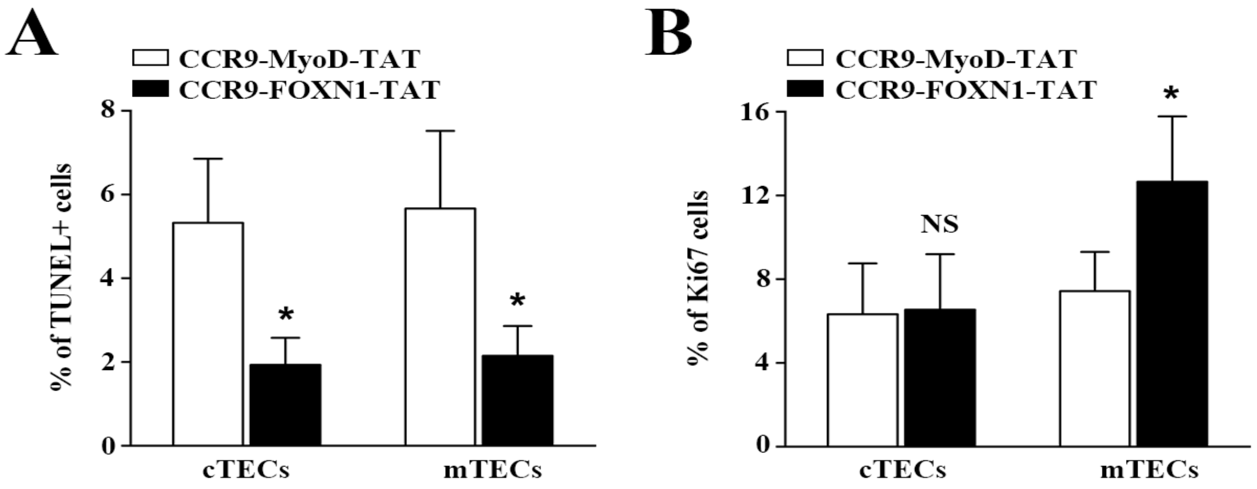

Supplemental Figure 5. rFOXN1 fusion protein reduces cell apoptosis of cTECs and mTECs and increases cell proliferation of mTECs in old mice. B6 mice (14-month-old) were injected i.v. with 80 µg rFOXN1 or control rMyoD fusion protein at 6 day-intervals as in Figure 3. Two months later, the thymi were harvested. The percentages of (A) $TUNEL^+$ apoptotic cells or (B) $Ki67^+$ proliferative cells in cTECs ($CD45^-EpCAM^+MHC\ II^+Ly51^+$), mTECs ($CD45^-EpCAM^+MHC\ II^+Ly51^-$) as analyzed by flow cytometry. Data are shown as mean $\pm$ SD (n = 6 mice/group) and are representative of three independent experiments with similar results. Statistical significance was determined by two-way ANOVA with post-hoc test. * p<0.05 compared with control rMyoD protein-treated mice.

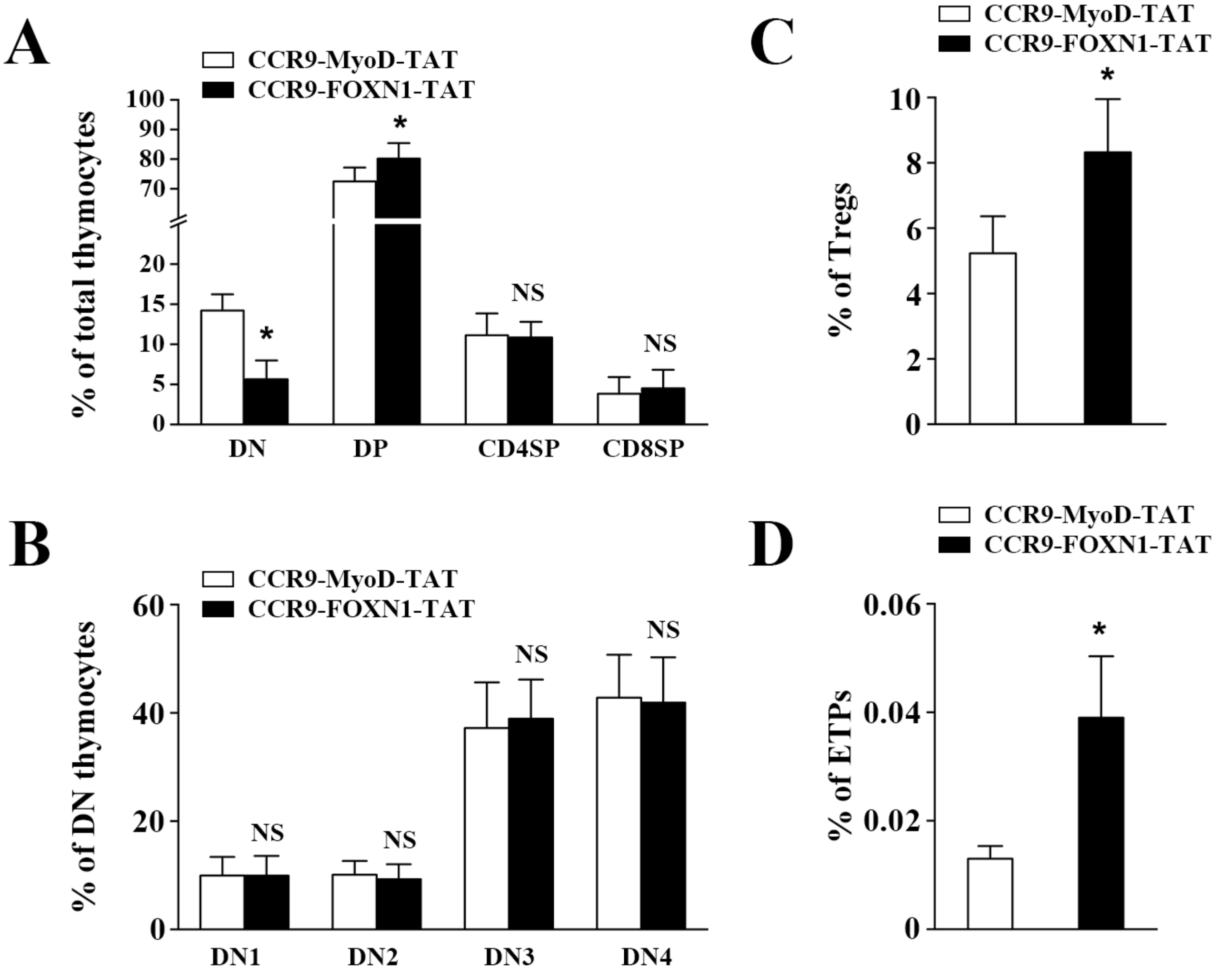

Supplemental Figure 6. The rFOXN1 fusion protein treatment affects the percentages of thymocyte subsets in old mice. B6 mice (14-month-old) were injected i.v. with 80 µg rFOXN1 or control rMyoD fusion protein at 6 day-intervals as in Figure 4. Two months later, the thymi were harvested and analyzed by flow cytometry. The percentages of (A) CD4 and CD8 DN, DP and CD4 or CD8 SP thymocytes, (B) DN1, DN2, DN3, and DN4 thymocytes after gating on lin^- cells, (C) $CD4^+CD25^+FoxP3^+$ Treg cells, and (D) $lin^-c\text{-}kit^+IL\text{-}7R\alpha^-CD44^+CD25^-$ ETPs in total thymocytes. Data are shown as mean ± SD (n = 8 mice/group) and are representative of three independent experiments with similar results. Statistical significance was determined by (A, B) two-way ANOVA with post-hoc test and (C, D) unpaired two-tailed Student's t-test. * $p<0.05$ compared with control rMyoD protein-treated mice.

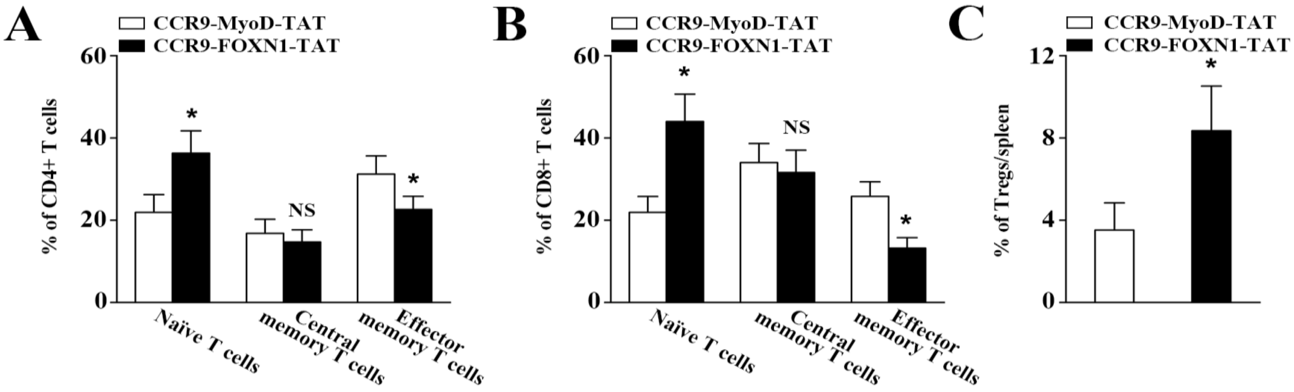

Supplemental Figure 7. The rFOXN1 fusion protein treatment affects the percentages of peripheral T cell subsets in old mice. B6 mice (14-month-old) were injected i.v. with 80 µg rFOXN1 or control rMyoD fusion protein at 6 day-intervals as in Figure 5. Two months later, the spleens were harvested and analyzed by flow cytometry. The percentages (A) CD4 and (B) CD8 (A, B) $CD44^{lo}CD62L^{hi}$ naïve, $CD44^{hi}CD62L^{lo}$ effector memory, and $CD44^{hi}CD62L^{hi}$ central memory T cells, and (C) $CD4^{+}CD25^{+}FoxP3^{+}$ Tregs. The data are shown as mean $\pm$ SD (n = 8 mice/group) and are representative of three independent experiments with similar results. Statistical significance was determined by (A, B) two-way ANOVA with post-hoc test and (C) unpaired two-tailed Student's t-test. * p<0.05 compared with control rMyoD protein-treated mice.

2 Supplementary Tables

Supplemental Table 1. Primers for qRT-PCR

Gene	Primer	Sequence 5'-3'
ccl 25	Forward	TTACCAGCACAGGATCAAATGG
	Reverse	CGGAAGTAGAATCTCACAGCAC
cxcl 12	Forward	TGCATCAGTGACGGTAAACCA
	Reverse	TTCTTCAGCCGTGCAACAATC
Dll4	Forward	CAGAGACTTCGCCAGGAAAC
	Reverse	ACTGCAGATGACCCGGTAAG
GAPDH	Forward	AGCATACAGGTCCTGGCATCTTGT
	Reverse	TGTAGACCATGTAGTTGAGGTCA
